# Bis(4-fluoro­benz­yl)bis­(4-phenyl-5-sulfanyl­idene-4,5-dihydro-1,3,4-thio­diazole-2-thiol­ato)tin(IV)

**DOI:** 10.1107/S1600536811054274

**Published:** 2011-12-23

**Authors:** Lei Li, Suyuan Zeng, Nana Yan

**Affiliations:** a College of Chemistry and Chemical Engineering, Liaocheng University, Shandong 252059, People’s Republic of China

## Abstract

In the title complex, [Sn(C_7_H_6_F)_2_(C_8_H_5_N_2_S_3_)_2_], including the weak Sn—N inter­actions, the Sn^IV^ atom is situated in a distorted *trans*-octa­hedral geometry, and the equatorial plane is defined by two chelating 4-phenyl-5-sulfanyl­idene-4,5-dihydro-1,3,4-thio­diazole-2-thiol­ate ligands. The apical positions are occupied by two C atoms of 4-fluoro­benzyl groups.

## Related literature

For related diorganotin(IV) 2-mercapto-4-methyl­pyrimidine derivatives, see: Ma *et al.* (2005 ▶).
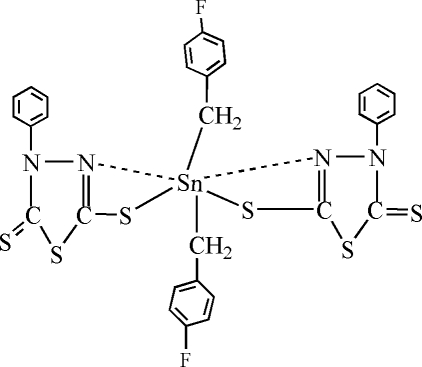


## Experimental

### 

#### Crystal data

[Sn(C_7_H_6_F)_2_(C_8_H_5_N_2_S_3_)_2_]
*M* *_r_* = 787.57
Triclinic, 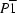

*a* = 10.856 (1) Å
*b* = 12.5901 (13) Å
*c* = 13.3741 (15) Å
α = 80.278 (2)°
β = 66.686 (1)°
γ = 77.918 (1)°
*V* = 1634.0 (3) Å^3^
*Z* = 2
Mo *K*α radiation
μ = 1.21 mm^−1^
*T* = 298 K
0.10 × 0.08 × 0.05 mm

#### Data collection

Siemens SMART CCD area-detector diffractometer
Absorption correction: multi-scan (*SADABS*; Sheldrick, 1996 ▶) *T*_min_ = 0.889, *T*_max_ = 0.942
8703 measured reflections
5687 independent reflections
2469 reflections with *I* > 2σ(*I*)
*R*_int_ = 0.068

#### Refinement

*R*[*F* ^2^ > 2σ(*F* ^2^)] = 0.060
*wR*(*F* ^2^) = 0.102
*S* = 0.83
5687 reflections
388 parameters
6 restraints
H-atom parameters constrained
Δρ_max_ = 0.53 e Å^−3^
Δρ_min_ = −0.57 e Å^−3^

### 

Data collection: *SMART* (Siemens, 1996 ▶); cell refinement: *SAINT* (Siemens, 1996 ▶); data reduction: *SAINT*; program(s) used to solve structure: *SHELXS97* (Sheldrick, 2008 ▶); program(s) used to refine structure: *SHELXL97* (Sheldrick, 2008 ▶); molecular graphics: *SHELXTL* (Sheldrick, 2008 ▶); software used to prepare material for publication: *SHELXTL*.

## Supplementary Material

Crystal structure: contains datablock(s) I, global. DOI: 10.1107/S1600536811054274/hp2022sup1.cif

Structure factors: contains datablock(s) I. DOI: 10.1107/S1600536811054274/hp2022Isup2.hkl

Additional supplementary materials:  crystallographic information; 3D view; checkCIF report

## Figures and Tables

**Table:** 

Sn1—C17	2.118 (7)
Sn1—C24	2.134 (6)
Sn1—S5	2.482 (2)
Sn1—S2	2.493 (2)

**Table:** 

C17—Sn1—C24	133.4 (3)
C17—Sn1—S5	109.1 (2)
C24—Sn1—S5	104.6 (2)
C17—Sn1—S2	103.5 (2)

**Fig. 1.**
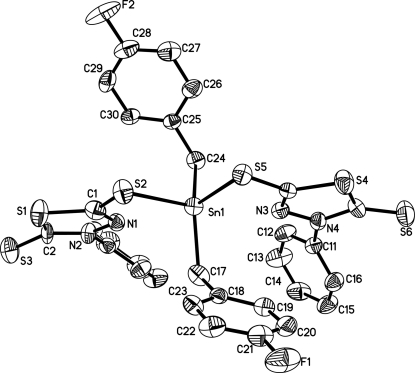
The molecular structure of the compound, showing 30% probability displacement ellipsoids.

## supplementary crystallographic information

### Comment

In the title compound, from Fig.1, as far as the weak Sn—N interactions are concerned, The coordination geometry of the Sn(IV) atom can be described as distorted *trans*-octahedral octahedral, with the basal plane defined by two symmertrically chelating 3-methylmercapto-5-mercapto-1,2,4-thiadiazole ligands. The apical positions are occupied by two 4-fluorobenzyl groups. The molecular structure consists of a monomer with a hexa-coordinated Sn atom surrounded by two S atoms and two N atoms of the ligand, and two 4-fluorobenzyl groups.

The Sn—S bond distances (Sn(1)—S(2)2.493 (2)Å and Sn(1)—S(5)2.482 (2) Å); and weak Sn—N bond lengths (Sn(1)—N(1)2.751Å and Sn(1)—N(3)2.688 Å) are close to those of the reported diorganotin(IV) 2-Mercapto-4-methylpyrimidine derivatives (Ma *et al.*, 2005). There is a good correspondence in their structure parameters: the Sn—S distances lie in the range 2.477–2.526Å and the Sn—N distances in the range 2.650–2.933 Å.

### Experimental

The mixture of the kalium salt of 2,5-dimercapto-4-phenyl-1,3,4-thiodiazole (2 mmol) was added to the solution of ethanol 20 ml, then add di(4-fluorobenzyl)tin(IV) dichloride(1 mmol) to the mixture, continuing the reaction for 12 h at 318k. After cooling down to room temperature, filtered it. The solvent of the filtrate was gradually removed by evaporation under vacuum until solid product was obtained. The solid was then recrystallized from ether-dichloromethane and colorless crystals suitable for X-ray diffraction were obtained (m.p. 433 K). Analysis, calculated for C_30_H_22_N_4_S_6_F_2_Sn: C 45.71, H 2.79,7.07; found: C 45.75, H 2.82,7.11%.

### Refinement

All H atoms were placed geometrically and treated as riding on their parent atoms with C—H 0.96 Å [*U*_iso_(H) = 1.2*U*_eq_(C)].

### Crystal data

**Table:**

[Sn(C_7_H_6_F)_2_(C_8_H_5_N_2_S_3_)_2_]	*Z* = 2
*M**_r_* = 787.57	*F*(000) = 788
Triclinic, *P*1	*D*_x_ = 1.601 Mg m^−^^3^
*a* = 10.856 (1) Å	Mo *K*α radiation, λ = 0.71073 Å
*b* = 12.5901 (13) Å	Cell parameters from 1083 reflections
*c* = 13.3741 (15) Å	θ = 2.5–25.2°
α = 80.278 (2)°	µ = 1.21 mm^−^^1^
β = 66.686 (1)°	*T* = 298 K
γ = 77.918 (1)°	Block, colorless
*V* = 1634.0 (3) Å^3^	0.10 × 0.08 × 0.05 mm

### Data collection

**Table:**

Siemens SMART CCD area-detector diffractometer	5687 independent reflections
Radiation source: fine-focus sealed tube	2469 reflections with *I* > 2σ(*I*)
graphite	*R*_int_ = 0.068
phi and ω scans	θ_max_ = 25.0°, θ_min_ = 1.7°
Absorption correction: multi-scan (*SADABS*; Sheldrick, 1996)	*h* = −12→12
*T*_min_ = 0.889, *T*_max_ = 0.942	*k* = −11→14
8703 measured reflections	*l* = −12→15

### Refinement

**Table:**

Refinement on *F*^2^	Primary atom site location: structure-invariant direct methods
Least-squares matrix: full	Secondary atom site location: difference Fourier map
*R*[*F*^2^ > 2σ(*F*^2^)] = 0.060	Hydrogen site location: inferred from neighbouring sites
*wR*(*F*^2^) = 0.102	H-atom parameters constrained
*S* = 0.83	*w* = 1/[σ^2^(*F*_o_^2^) + (0.0195*P*)^2^] where *P* = (*F*_o_^2^ + 2*F*_c_^2^)/3
5687 reflections	(Δ/σ)_max_ < 0.001
388 parameters	Δρ_max_ = 0.53 e Å^−^^3^
6 restraints	Δρ_min_ = −0.57 e Å^−^^3^

### Special details

**Table:**

Geometry. All e.s.d.'s (except the e.s.d. in the dihedral angle between two l.s. planes) are estimated using the full covariance matrix. The cell e.s.d.'s are taken into account individually in the estimation of e.s.d.'s in distances, angles and torsion angles; correlations between e.s.d.'s in cell parameters are only used when they are defined by crystal symmetry. An approximate (isotropic) treatment of cell e.s.d.'s is used for estimating e.s.d.'s involving l.s. planes.
Refinement. Refinement of *F*^2^ against ALL reflections. The weighted *R*-factor *wR* and goodness of fit *S* are based on *F*^2^, conventional *R*-factors *R* are based on *F*, with *F* set to zero for negative *F*^2^. The threshold expression of *F*^2^ > σ(*F*^2^) is used only for calculating *R*-factors(gt) *etc*. and is not relevant to the choice of reflections for refinement. *R*-factors based on *F*^2^ are statistically about twice as large as those based on *F*, and *R*- factors based on ALL data will be even larger.

### Fractional atomic coordinates and isotropic or equivalent isotropic displacement parameters (Å^2^)

**Table:**

	*x*	*y*	*z*	*U*_iso_*/*U*_eq_	
Sn1	0.62742 (6)	0.71890 (5)	0.14274 (5)	0.0496 (2)	
F1	0.1254 (5)	1.1462 (5)	0.2820 (4)	0.113 (2)	
F2	0.8752 (6)	0.4276 (5)	−0.2743 (5)	0.131 (3)	
N1	0.5853 (6)	0.5138 (6)	0.2456 (5)	0.0511 (18)	
N2	0.5920 (6)	0.4179 (6)	0.3132 (5)	0.0579 (19)	
N3	0.7364 (6)	0.8976 (6)	0.1249 (5)	0.0514 (19)	
N4	0.7996 (6)	0.9668 (6)	0.1511 (5)	0.0524 (18)	
S1	0.4427 (2)	0.3901 (2)	0.2215 (2)	0.0792 (8)	
S2	0.4857 (2)	0.6158 (2)	0.09849 (18)	0.0676 (7)	
S3	0.4882 (3)	0.2311 (2)	0.4009 (2)	0.0913 (9)	
S4	0.7446 (3)	1.0676 (2)	−0.00804 (19)	0.0741 (8)	
S5	0.6124 (2)	0.87078 (18)	0.00113 (17)	0.0572 (7)	
S6	0.8989 (3)	1.1587 (2)	0.0923 (2)	0.0930 (9)	
C1	0.5097 (8)	0.5101 (8)	0.1911 (7)	0.060 (3)	
C2	0.5175 (8)	0.3428 (7)	0.3176 (7)	0.060 (3)	
C3	0.6751 (8)	0.4109 (8)	0.3786 (7)	0.053 (2)	
C4	0.6719 (9)	0.5004 (8)	0.4251 (7)	0.071 (3)	
H4	0.6169	0.5659	0.4168	0.085*	
C5	0.7523 (9)	0.4908 (10)	0.4845 (7)	0.087 (3)	
H5	0.7510	0.5507	0.5174	0.104*	
C6	0.8326 (10)	0.3970 (11)	0.4961 (8)	0.090 (4)	
H6	0.8855	0.3917	0.5375	0.108*	
C7	0.8366 (10)	0.3092 (10)	0.4468 (9)	0.090 (4)	
H7	0.8921	0.2441	0.4554	0.108*	
C8	0.7601 (9)	0.3155 (8)	0.3850 (7)	0.076 (3)	
H8	0.7659	0.2569	0.3485	0.091*	
C9	0.6986 (7)	0.9418 (7)	0.0439 (6)	0.050 (2)	
C10	0.8221 (8)	1.0629 (8)	0.0864 (6)	0.058 (3)	
C11	0.8314 (8)	0.9337 (7)	0.2466 (6)	0.049 (2)	
C12	0.8946 (8)	0.8290 (8)	0.2593 (7)	0.067 (3)	
H12	0.9251	0.7835	0.2035	0.080*	
C13	0.9119 (9)	0.7929 (8)	0.3567 (8)	0.090 (4)	
H13	0.9538	0.7217	0.3670	0.108*	
C14	0.8697 (9)	0.8583 (9)	0.4367 (7)	0.081 (3)	
H14	0.8815	0.8320	0.5021	0.097*	
C15	0.8087 (9)	0.9644 (8)	0.4233 (7)	0.075 (3)	
H15	0.7829	1.0110	0.4776	0.090*	
C16	0.7871 (8)	0.9995 (7)	0.3285 (7)	0.066 (3)	
H16	0.7413	1.0695	0.3199	0.079*	
C17	0.5000 (7)	0.7694 (7)	0.2992 (6)	0.066 (3)	
H17A	0.4513	0.7106	0.3428	0.079*	
H17B	0.5559	0.7834	0.3355	0.079*	
C18	0.4001 (8)	0.8690 (7)	0.2947 (6)	0.048 (2)	
C19	0.4254 (8)	0.9709 (9)	0.3001 (6)	0.060 (3)	
H19	0.5040	0.9766	0.3096	0.072*	
C20	0.3345 (11)	1.0652 (8)	0.2915 (7)	0.074 (3)	
H20	0.3533	1.1337	0.2922	0.089*	
C21	0.2189 (11)	1.0542 (9)	0.2823 (7)	0.071 (3)	
C22	0.1870 (9)	0.9558 (10)	0.2815 (7)	0.072 (3)	
H22	0.1040	0.9506	0.2791	0.086*	
C23	0.2798 (9)	0.8647 (8)	0.2844 (6)	0.060 (3)	
H23	0.2612	0.7974	0.2793	0.072*	
C24	0.8311 (6)	0.6359 (6)	0.0774 (6)	0.053 (2)	
H24A	0.8936	0.6882	0.0534	0.063*	
H24B	0.8508	0.5843	0.1337	0.063*	
C25	0.8501 (7)	0.5773 (8)	−0.0164 (7)	0.045 (2)	
C26	0.8832 (7)	0.6322 (8)	−0.1193 (8)	0.063 (3)	
H26	0.8991	0.7038	−0.1302	0.075*	
C27	0.8930 (9)	0.5809 (9)	−0.2076 (8)	0.075 (3)	
H27	0.9151	0.6177	−0.2773	0.090*	
C28	0.8696 (9)	0.4764 (11)	−0.1896 (9)	0.074 (3)	
C29	0.8407 (7)	0.4194 (8)	−0.0905 (9)	0.065 (3)	
H29	0.8276	0.3471	−0.0807	0.078*	
C30	0.8313 (7)	0.4705 (8)	−0.0046 (7)	0.050 (2)	
H30	0.8115	0.4318	0.0641	0.060*	

### Atomic displacement parameters (Å^2^)

**Table:**

	*U*^11^	*U*^22^	*U*^33^	*U*^12^	*U*^13^	*U*^23^
Sn1	0.0464 (3)	0.0523 (5)	0.0507 (4)	−0.0017 (3)	−0.0227 (3)	−0.0029 (3)
F1	0.125 (5)	0.091 (5)	0.086 (4)	0.045 (4)	−0.031 (4)	−0.009 (3)
F2	0.132 (5)	0.163 (7)	0.117 (5)	0.043 (4)	−0.065 (4)	−0.101 (5)
N1	0.044 (4)	0.049 (5)	0.058 (5)	−0.008 (4)	−0.016 (4)	−0.007 (4)
N2	0.066 (5)	0.042 (5)	0.073 (5)	−0.012 (4)	−0.035 (4)	0.001 (4)
N3	0.053 (4)	0.051 (5)	0.048 (5)	−0.006 (4)	−0.016 (4)	−0.009 (4)
N4	0.057 (4)	0.051 (6)	0.053 (5)	−0.015 (4)	−0.021 (4)	−0.004 (4)
S1	0.0875 (18)	0.065 (2)	0.111 (2)	−0.0242 (16)	−0.0621 (17)	0.0032 (16)
S2	0.0679 (15)	0.072 (2)	0.0793 (18)	−0.0174 (14)	−0.0469 (14)	0.0075 (14)
S3	0.097 (2)	0.066 (2)	0.116 (2)	−0.0275 (16)	−0.0476 (18)	0.0145 (17)
S4	0.1010 (19)	0.064 (2)	0.0703 (17)	−0.0276 (16)	−0.0475 (16)	0.0165 (14)
S5	0.0689 (15)	0.0533 (17)	0.0574 (15)	−0.0083 (13)	−0.0358 (13)	0.0028 (12)
S6	0.128 (2)	0.078 (2)	0.090 (2)	−0.0497 (19)	−0.0509 (18)	0.0119 (16)
C1	0.045 (5)	0.072 (8)	0.062 (6)	−0.003 (5)	−0.022 (5)	−0.005 (5)
C2	0.057 (6)	0.040 (6)	0.087 (7)	−0.023 (5)	−0.025 (5)	−0.006 (5)
C3	0.048 (5)	0.047 (7)	0.053 (6)	−0.004 (5)	−0.010 (5)	−0.005 (5)
C4	0.077 (7)	0.060 (8)	0.074 (7)	0.010 (6)	−0.033 (6)	−0.019 (6)
C5	0.071 (7)	0.133 (12)	0.075 (7)	−0.002 (7)	−0.044 (6)	−0.035 (7)
C6	0.077 (8)	0.119 (12)	0.080 (8)	0.004 (8)	−0.046 (7)	−0.008 (8)
C7	0.082 (8)	0.096 (11)	0.097 (9)	0.011 (7)	−0.052 (7)	−0.005 (7)
C8	0.073 (7)	0.068 (8)	0.082 (7)	0.016 (6)	−0.035 (6)	−0.014 (6)
C9	0.055 (5)	0.053 (6)	0.037 (5)	−0.014 (5)	−0.010 (4)	−0.005 (4)
C10	0.054 (5)	0.061 (7)	0.054 (6)	−0.022 (5)	−0.016 (5)	0.009 (5)
C11	0.044 (5)	0.055 (7)	0.051 (6)	−0.006 (5)	−0.022 (5)	−0.009 (5)
C12	0.068 (6)	0.076 (8)	0.061 (7)	0.005 (6)	−0.031 (5)	−0.021 (6)
C13	0.101 (9)	0.094 (9)	0.072 (8)	0.034 (7)	−0.050 (7)	−0.015 (7)
C14	0.091 (8)	0.090 (9)	0.050 (7)	0.010 (7)	−0.032 (6)	0.005 (6)
C15	0.103 (8)	0.073 (8)	0.056 (7)	−0.006 (6)	−0.037 (6)	−0.015 (6)
C16	0.078 (7)	0.052 (7)	0.066 (7)	0.000 (5)	−0.030 (6)	−0.007 (6)
C17	0.053 (5)	0.075 (8)	0.065 (6)	−0.008 (5)	−0.025 (5)	0.007 (5)
C18	0.050 (6)	0.042 (6)	0.040 (5)	−0.004 (5)	−0.008 (4)	−0.001 (4)
C19	0.057 (6)	0.067 (8)	0.053 (6)	−0.008 (6)	−0.017 (5)	−0.008 (5)
C20	0.091 (8)	0.054 (8)	0.063 (7)	−0.008 (7)	−0.014 (6)	−0.008 (5)
C21	0.078 (8)	0.063 (9)	0.056 (6)	0.012 (7)	−0.022 (6)	0.004 (6)
C22	0.055 (6)	0.095 (10)	0.069 (7)	0.002 (7)	−0.028 (5)	−0.021 (6)
C23	0.048 (6)	0.066 (7)	0.059 (6)	−0.014 (5)	−0.014 (5)	0.000 (5)
C24	0.033 (5)	0.063 (7)	0.063 (6)	−0.003 (4)	−0.020 (4)	−0.008 (5)
C25	0.035 (5)	0.054 (7)	0.045 (6)	0.000 (5)	−0.014 (5)	−0.011 (5)
C26	0.054 (6)	0.061 (7)	0.068 (7)	0.004 (5)	−0.018 (6)	−0.020 (6)
C27	0.075 (7)	0.079 (9)	0.065 (7)	0.001 (7)	−0.024 (6)	−0.011 (6)
C28	0.058 (7)	0.100 (11)	0.070 (8)	0.012 (7)	−0.028 (6)	−0.042 (8)
C29	0.041 (5)	0.045 (7)	0.107 (9)	0.007 (5)	−0.022 (6)	−0.034 (7)
C30	0.051 (5)	0.043 (7)	0.050 (6)	0.011 (5)	−0.019 (5)	−0.010 (5)

### Geometric parameters (Å, °)

**Table:**

Sn1—C17	2.118 (7)	C12—C13	1.379 (10)
Sn1—C24	2.134 (6)	C12—H12	0.9300
Sn1—S5	2.482 (2)	C13—C14	1.336 (11)
Sn1—S2	2.493 (2)	C13—H13	0.9300
F1—C21	1.372 (10)	C14—C15	1.377 (11)
F2—C28	1.352 (10)	C14—H14	0.9300
N1—C1	1.307 (9)	C15—C16	1.365 (10)
N1—N2	1.388 (8)	C15—H15	0.9300
N2—C2	1.347 (9)	C16—H16	0.9300
N2—C3	1.467 (10)	C17—C18	1.486 (10)
N3—C9	1.305 (8)	C17—H17A	0.9700
N3—N4	1.376 (8)	C17—H17B	0.9700
N4—C10	1.370 (9)	C18—C23	1.379 (10)
N4—C11	1.426 (8)	C18—C19	1.387 (10)
S1—C1	1.731 (9)	C19—C20	1.395 (11)
S1—C2	1.741 (9)	C19—H19	0.9300
S2—C1	1.711 (9)	C20—C21	1.345 (11)
S3—C2	1.642 (8)	C20—H20	0.9300
S4—C9	1.711 (8)	C21—C22	1.356 (12)
S4—C10	1.761 (8)	C22—C23	1.366 (11)
S5—C9	1.717 (8)	C22—H22	0.9300
S6—C10	1.631 (9)	C23—H23	0.9300
C3—C4	1.364 (11)	C24—C25	1.485 (10)
C3—C8	1.365 (10)	C24—H24A	0.9700
C4—C5	1.370 (11)	C24—H24B	0.9700
C4—H4	0.9300	C25—C26	1.375 (10)
C5—C6	1.340 (12)	C25—C30	1.375 (10)
C5—H5	0.9300	C26—C27	1.397 (11)
C6—C7	1.364 (13)	C26—H26	0.9300
C6—H6	0.9300	C27—C28	1.355 (13)
C7—C8	1.368 (11)	C27—H27	0.9300
C7—H7	0.9300	C28—C29	1.348 (12)
C8—H8	0.9300	C29—C30	1.368 (11)
C11—C16	1.357 (10)	C29—H29	0.9300
C11—C12	1.368 (10)	C30—H30	0.9300
			
C17—Sn1—C24	133.4 (3)	C13—C14—C15	120.7 (8)
C17—Sn1—S5	109.1 (2)	C13—C14—H14	119.7
C24—Sn1—S5	104.6 (2)	C15—C14—H14	119.7
C17—Sn1—S2	103.5 (2)	C16—C15—C14	118.5 (8)
C24—Sn1—S2	106.2 (2)	C16—C15—H15	120.8
S5—Sn1—S2	92.46 (8)	C14—C15—H15	120.8
C1—N1—N2	110.3 (7)	C11—C16—C15	120.9 (8)
C2—N2—N1	118.2 (7)	C11—C16—H16	119.6
C2—N2—C3	125.8 (7)	C15—C16—H16	119.6
N1—N2—C3	115.9 (7)	C18—C17—Sn1	113.1 (5)
C9—N3—N4	111.2 (7)	C18—C17—H17A	109.0
C10—N4—N3	117.8 (7)	Sn1—C17—H17A	109.0
C10—N4—C11	125.4 (7)	C18—C17—H17B	109.0
N3—N4—C11	116.7 (7)	Sn1—C17—H17B	109.0
C1—S1—C2	91.1 (4)	H17A—C17—H17B	107.8
C1—S2—Sn1	90.0 (3)	C23—C18—C19	117.2 (8)
C9—S4—C10	91.6 (4)	C23—C18—C17	122.1 (9)
C9—S5—Sn1	89.7 (3)	C19—C18—C17	120.8 (9)
N1—C1—S2	121.0 (7)	C18—C19—C20	121.0 (9)
N1—C1—S1	113.4 (7)	C18—C19—H19	119.5
S2—C1—S1	125.6 (6)	C20—C19—H19	119.5
N2—C2—S3	129.2 (7)	C21—C20—C19	118.2 (10)
N2—C2—S1	106.8 (6)	C21—C20—H20	120.9
S3—C2—S1	123.8 (5)	C19—C20—H20	120.9
C4—C3—C8	122.1 (9)	C20—C21—C22	122.9 (10)
C4—C3—N2	119.7 (8)	C20—C21—F1	118.0 (11)
C8—C3—N2	118.0 (9)	C22—C21—F1	118.9 (11)
C3—C4—C5	117.9 (9)	C21—C22—C23	118.3 (9)
C3—C4—H4	121.0	C21—C22—H22	120.9
C5—C4—H4	121.0	C23—C22—H22	120.9
C6—C5—C4	121.4 (11)	C22—C23—C18	122.3 (9)
C6—C5—H5	119.3	C22—C23—H23	118.8
C4—C5—H5	119.3	C18—C23—H23	118.8
C5—C6—C7	119.7 (11)	C25—C24—Sn1	110.3 (5)
C5—C6—H6	120.1	C25—C24—H24A	109.6
C7—C6—H6	120.1	Sn1—C24—H24A	109.6
C6—C7—C8	121.0 (10)	C25—C24—H24B	109.6
C6—C7—H7	119.5	Sn1—C24—H24B	109.6
C8—C7—H7	119.5	H24A—C24—H24B	108.1
C3—C8—C7	117.8 (10)	C26—C25—C30	117.9 (8)
C3—C8—H8	121.1	C26—C25—C24	119.3 (9)
C7—C8—H8	121.1	C30—C25—C24	122.7 (8)
N3—C9—S4	113.6 (6)	C25—C26—C27	120.3 (10)
N3—C9—S5	118.8 (7)	C25—C26—H26	119.8
S4—C9—S5	127.6 (5)	C27—C26—H26	119.8
N4—C10—S6	129.8 (7)	C28—C27—C26	118.7 (10)
N4—C10—S4	105.6 (6)	C28—C27—H27	120.7
S6—C10—S4	124.6 (5)	C26—C27—H27	120.7
C16—C11—C12	120.4 (7)	C29—C28—F2	118.7 (12)
C16—C11—N4	121.0 (8)	C29—C28—C27	122.5 (10)
C12—C11—N4	118.2 (8)	F2—C28—C27	118.8 (11)
C11—C12—C13	118.4 (8)	C28—C29—C30	118.3 (10)
C11—C12—H12	120.8	C28—C29—H29	120.8
C13—C12—H12	120.8	C30—C29—H29	120.8
C14—C13—C12	121.1 (8)	C29—C30—C25	122.2 (8)
C14—C13—H13	119.5	C29—C30—H30	118.9
C12—C13—H13	119.5	C25—C30—H30	118.9
